# Effects of Dietary Fibers with Different Physicochemical Properties on Intestinal Health and Growth Performance of Meat Rabbits

**DOI:** 10.3390/ani16182921

**Published:** 2026-09-17

**Authors:** Yeqiu Zhang, Ying Wang, Shiting Xiao, Si Wang, Kangle Li, Qinghua Liu, Shuangbo Huang

**Affiliations:** 1 Engineering Research Center for Animal Breeding and Sustainable Production, College of Animal Sciences, Fujian Agriculture and Forestry University, Fuzhou 350002, China; 52306009005@fafu.edu.cn (Y.Z.); 52406009017@fafu.edu.cn (Y.W.); 52506009017@fafu.edu.cn (S.X.); 12506009010@fafu.edu.cn (S.W.); likanglesoon@163.com (K.L.); 2 Key Laboratory of Animal Pathogen Infection and Immunology of Fujian Province, College of Animal Sciences, Fujian Agriculture and Forestry University, Fuzhou 350002, China

**Keywords:** fiber raw materials, diarrhea, growth performance, hydration property, fermentation, principal component analysis, meat rabbits, short-chain fatty acid

## Abstract

Dietary fiber raw materials for meat rabbits are diverse, yet the effects of their hydration properties and fermentation on growth performance and intestinal health remain insufficiently understood. In this study, we first systematically analyzed 12 commercial fiber raw materials for nutrient composition, hydration properties, and in vitro fermentation characteristics. Principal component analysis (PCA) was employed to reduce the dimensionality of the nutritional and physicochemical parameters, extracting two key dimensions: the first was predominantly defined by fiber fractions and viscosity, while the second was jointly driven by water binding capacity (WBC), water swelling capacity (WSC), gas production, and volatile fatty acid (VFA) generation. Subsequently, based on the similarity of fibers along the first principal component and their gradient distribution along the second, four representative fiber raw materials with graded hydration properties and fermentation were selected for in vivo validation: peanut vine meal (PV) < *Pennisetum giganteum* meal (PG) < *Moringa oleifera* leaf meal (MO) < mulberry leaf meal (ML). A total of 96 weaned New Zealand white rabbits were randomly assigned to four groups and fed diets containing PV, PG, MO, or ML, respectively, over a 35-day feeding trial. Growth performance, diarrhea incidence, intestinal morphology, cecal microbiota, and VFA profiles were evaluated. In vivo results showed that MO was associated with optimized cecal microbiota, increased short-chain fatty acids, and reduced diarrhea. In contrast, despite its high in vitro fermentation, ML was associated with intestinal inflammation, decreased short-chain fatty acids, reduced average daily gain and average daily feed intake, elevated branched-chain fatty acids and ammonia nitrogen, and increased diarrhea incidence. Correlation analysis further revealed that enriched *Bacteroides* was closely associated with reduced growth performance and impaired intestinal health. Collectively, these findings suggest that MO with moderate hydration properties and high fermentation may be more beneficial for optimizing growth performance and intestinal health in meat rabbits than ML with high fermentation alone. This study provides a reference for the rational selection of fiber resources in meat rabbit production.

## Simple Summary

The type of dietary fiber in rabbit feed can greatly influence gut health and growth, but not all fiber raw materials are equally beneficial. In this study, we first measured the nutritional value, hydration properties and fermentation of 12 different fiber raw materials. Based on these measurements, we selected four fiber raw materials with increasing hydration properties and fermentation, namely peanut vine meal, *Pennisetum giganteum* meal, *Moringa oleifera* leaf meal, and mulberry leaf meal, and fed them to 96 weaned rabbits over 35 days. Results showed that Moringa oleifera leaf meal improved gut bacteria, increased beneficial fermentation products, and reduced diarrhea. In contrast, mulberry leaf meal, despite being highly fermentable, was associated with gut inflammation, higher ammonia nitrogen levels, reduced growth performance, and increased diarrhea in the rabbits. Our findings provide practical guidance for feed manufacturers and farmers in choosing better fiber raw materials for meat rabbit diets.

## 1. Introduction

Weaning in meat rabbits represents a critical transitional stage, marked by abrupt dietary shifts, immature gastrointestinal function, and heightened vulnerability to enteric disorders such as diarrhea, ultimately leading to stunted growth and increased mortality rates [1]. In commercial rabbit production, dietary fiber is no longer merely a bulking agent but a key functional component that modulates gut health, yet not all fiber raw materials are equally suited to the fragile digestive system of newly weaned rabbits [2]. While the crude fiber content is routinely listed in feed formulations, growing evidence indicates that the functional properties of fiber raw materials—particularly hydration and fermentation characteristics—exert a more pronounced influence on intestinal physiology and growth performance than their chemical composition alone [3]. Hydration-related indicators, including viscosity, water binding capacity (WBC) and water swelling capacity (WSC), directly regulate digesta transit rate in the digestive tract. High-viscosity or excessively hydrated fibers can slow digest transit, impede the interaction between digestive enzymes and substrates, and disrupt microbial equilibrium, thereby elevating the risk of diarrhea [4]. Conversely, fibers with moderate hydration profiles support controlled hindgut fermentation, promote the proliferation of beneficial bacteria, and maintain the integrity of the intestinal barrier [5]. The fermentation of dietary fibers by the cecal microbiota yields short-chain fatty acids (SCFAs), primarily acetate, propionate, and butyrate, which serve as major energy sources for colonocytes, modulate local and systemic immune responses, and reinforce intestinal barrier function. However, current studies on rabbit nutrition mainly focus on the supplemental dosage of dietary fiber, while few studies have systematically elucidated how the interplay between hydration properties and the fermentation of various fiber raw materials coordinately regulates nutrient utilization and intestinal health in weaned meat rabbits, leaving a prominent research gap [2].

In vitro fermentation assays, which are widely used to rank fiber fermentation, may not reliably predict in vivo outcomes. In these assays, fibers are finely ground and suspended in buffered media, allowing unimpeded microbial access to substrates, which are conditions that maximize fermentation potential but do not replicate the physical and dynamic environment of the living gut [6]. In intact animals, factors such as digesta viscosity, WBC, WSC, transit rate, and the three-dimensional structure of the fiber matrix can substantially modify the actual fermentation profile [4,7]. Consequently, a fiber that appears highly fermentable in vitro may, in vivo, provoke proteolytic fermentation, generate excessive ammonia nitrogen (NH_3_-N) and branched-chain fatty acids (BCFAs), and even impair intestinal barrier function, as has been observed in some studies with high hydration fibers [8,9]. Despite this recognized discrepancy, direct comparisons between in vitro fermentation indices and in vivo physiological responses within the same experimental framework remain scarce, especially for fiber raw materials commonly used in rabbit production.

Accordingly, we hypothesized that dietary fibers with distinct physicochemical gradients (hydration and fermentation) would exert differential regulatory effects on the growth performance, diarrhea incidence and cecal microbiota composition of weaned New Zealand white rabbits. To verify this hypothesis, we first determined the in vitro physicochemical indicators of 12 common fiber raw materials, and four typical fibers with differentiated physicochemical gradients were selected for animal feeding trials. This study aimed to clarify the internal mechanism by which fiber hydration and fermentation synergistically regulate intestinal health in weaned meat rabbits, screen high-quality fiber materials suitable for meat rabbit diets, and provide theoretical basis and practical references for the precise selection of dietary fibers in meat rabbit feed formulation.

## 2. Materials and Methods

### 2.1. Fiber Raw Materials

In this study, 12 fiber raw materials from different sources were selected as the test subjects, all purchased from commercial feed suppliers. Detailed supplier information and batch numbers for each fiber are provided in Supplementary Table S1. The 12 fiber raw materials in this study were selected according to predefined criteria, including potential use, botanical origin, commercial availability in local feed markets, and regional agricultural characteristics of South China [10]. Sodium alginate and konjac flour are typical colloidal fiber materials, while kelp fiber is a representative marine-derived fiber [11,12,13]. Garlic peel, soybean straw, peanut vine (PV) and bamboo fiber are agricultural by-product fibers [14]. Paper mulberry leaf meal, *Moringa oleifera* leaf meal (MO), mulberry leaf meal (ML), and *Pennisetum giganteum* meal (PG) are characteristic feed resources widely cultivated in South China [10,15]. Alfalfa meal serves as a conventional fiber material [16]. All samples were pulverized and sieved through a 40-mesh sieve, then stored in a room temperature desiccator for subsequent analysis. For each fiber type, representative samples were obtained from four independent batches (n = four batches per fiber type). Each sample was analyzed in duplicate (technical replicates), and the mean value of the two replicates was used as the batch-specific value. Data are presented as the mean of the four independent batches for descriptive comparison among fiber types.

### 2.2. Determination of Basic Characteristics of Fiber Raw Materials and Experimental Diets

Dry matter (DM) content was measured by oven-drying samples to a constant weight at 103 °C (AOAC 930.15). Crude protein (CP) content was quantified via the Kjeldahl method (AOAC 984.13), with a nitrogen-to-protein conversion factor of 6.25. Crude fiber (CF) content was determined using the acid–alkali digestion procedure (AOAC 978.10). Neutral detergent fiber (NDF) and acid detergent fiber (ADF) contents were sequentially quantified with an Ankom 200 Fiber Analyzer (Ankom Technology, Macedon, NY, USA) following the protocol of Van Soest et al. [17]. Acid detergent lignin (ADL) was determined by the 72% sulfuric acid digestion method (AOAC 973.18). Soluble dietary fiber (SDF) was analyzed using the enzymatic–gravimetric method (AOAC 991.43). Each measurement was conducted in triplicate.

### 2.3. Hydration Properties of Different Fiber Raw Material

The hydration properties of dietary fibers include WBC, WSC, and viscosity.

WBC was determined in accordance with the method established by Ma et al. [18]. Briefly, fiber samples were oven-dried to constant weight at 105 °C. Approximately 0.3 g of dried fiber sample was accurately transferred into a 50 mL centrifuge tube, to which 10 mL of distilled water was added. The mixture was equilibrated at ambient temperature for 1 h and then centrifuged at 14,000× *g* for 20 min. After centrifugation, the supernatant was carefully decanted. The centrifuge tube with residual sediment was drained for 30 min and weighed. Afterwards, the sediment was oven-dried again to constant weight at 105 °C and reweighed. The weight difference between the two measurements corresponded to the amount of water bound to the fiber matrix.

WSC was assayed following the protocol published by Huang et al. [8,19]. Approximately 0.2 g of dried fiber sample (W_0_) was accurately weighed into a graduated test tube, and the initial volume of the dry sample (V_0_) was recorded. Then, a total of 10 mL distilled water was added, and the blend was fully homogenized before being equilibrated at room temperature for 18 h. The final volume of the water-saturated swollen fiber (V_1_) was recorded. The WSC (mL/g) was calculated via the formula below: WSC = (V_1_ − V_0_)/W_0_.

Viscosity was measured using the procedure reported by Tan et al. [20]. Approximately 2 g of fiber sample was weighed into a 15 mL centrifuge tube, and mixed with 10 mL of 0.9% NaCl solution (Sinopharm Chemical Reagent Co., Ltd., Shanghai, China). The suspension was incubated in a water bath at 40 °C for 1 h, followed by centrifugation at 4 °C for 25 min. Subsequently, 0.5 mL of the supernatant was carefully withdrawn using a pipette for viscosity analysis. Shear viscosity was measured in a Brookfield DV-11 cone/plate viscometer (Brookfield Engineering Laboratories Inc., Middleboro, MA, USA), and all measurements were performed at a constant temperature of 38 °C, with shear rates ranging from 2.25 to 450 s^−1^ [20]. The final reported viscosity value represents the average obtained at shear rates of 225, 240, 255, 270, 285, and 300 s^−1^.

### 2.4. In Vitro Fermentation Characteristics of Different Fiber Raw Materials

Cecal inoculum was collected from New Zealand White rabbits aged 45 ± 2 day. Six healthy, weight-matched rabbits were fasted for 12 h (with water provided ad libitum) prior to slaughter to minimize inter-individual variation in the cecal microbiota and provide sufficient volume for all treatment bottles. Fresh cecal digesta were aseptically harvested, pooled, and processed according to previously established protocols [21]. Briefly, the pooled digesta was diluted 1:4 (*w*/*v*) with prewarmed 0.9% NaCl solution (Sinopharm Chemical Reagent Co., Ltd., Shanghai, China), thoroughly homogenized, and filtered through four layers of sterile cheesecloth to obtain the inoculum filtrate. The filtrate was then mixed with fermentation medium at a 1:2 (*v*/*v*) ratio. For each fiber sample, two independent fermentation bottles were prepared, and each bottle was treated as one technical replicate. Approximately 0.25 g of dietary substrate was accurately weighed into each fermentation bottle, followed by the addition of 75 mL of the inoculum–medium mixture. The inoculum-to-substrate ratio was standardized at approximately 300 mL of the inoculum–medium mixture per gram of substrate for all samples, based on the fixed substrate weight (0.25 g) and fixed volume (75 mL). Blank bottles containing the inoculum–medium mixture without substrate were included in each run to correct for background gas production and baseline SCFAs and NH_3_-N concentrations originating from the inoculum. Continuous CO_2_ flushing was maintained throughout the incubation to ensure strictly anaerobic conditions. Fermentation was conducted at 39 °C for 48 h using an ANKOM automated gas production recording system (Ankom Technology, Macedon, NY, USA). Net gas production for each substrate bottle was calculated by subtracting the gas volume recorded in the corresponding blank bottle at each time point, and is expressed as mL per gram of substrate on an as-fed basis. At the end of the 48 h incubation period, fermentation was terminated. Supernatant aliquots were collected and stored at −20 °C for the subsequent analysis of NH_3_-N and SCFAs, and the concentrations, corrected by subtracting blank values, were reported as μg per mL of fermentation broth. NH_3_-N concentration was determined spectrophotometrically using the salicylic acid–sodium hypochlorite method [8]. A standard stock solution was prepared by dissolving 0.382 g of ammonium chloride in 0.2 mol/L HCl and diluting to 100 mL; the stock solution was stored at 4 °C in the dark. Working standards were prepared by serial dilution of the stock solution immediately before color development. Two reagents were freshly prepared: Reagent A [0.08 g sodium nitroprusside (Sinopharm Chemical Reagent Co., Ltd., Shanghai, China) dissolved in 100 mL of 14% *w*/*v* sodium salicylate (Sinopharm Chemical Reagent Co., Ltd., Shanghai, China)] and Reagent B [2 mL commercial sodium hypochlorite solution (Sigma-Aldrich, St. Louis, MO, USA) diluted to 100 mL with 0.3 mol/L NaOH (Sigma-Aldrich, St. Louis, MO, USA)]. For both standards and sample supernatants, 0.4 mL of each solution was mixed with 2 mL of Reagent A and 2 mL of Reagent B, and incubated for 10 min at ambient temperature; the absorbance was measured at 700 nm against a reagent blank to quantify NH_3_-N.

### 2.5. Kinetic Analysis of In Vitro Gas Production

Gas production parameters were fitted using the nonlinear software program NLREG v6.5 (Phillip H. Sherrod, Nashville, TN, USA) according to the logistic-exponential equation proposed by Tan et al. [22]: V = V_F_ × (1 − exp(−K × T))/(1 + exp(B − K × T)), where V_F_ is the theoretical maximum gas production (mL/g), K is the fermentation rate constant (h^−1^), B is the shape parameter of the gas production curve, and T is the fermentation time (h). From these fitted values, two additional parameters were derived: initial gas production rate (IR0) and half-life to asymptote (T½) [22].

### 2.6. Experimental Design of the Rabbit Feeding Trial

A total of 96 weaned New Zealand white rabbits aged 35 ± 2 days (half male and half female) was allotted into four dietary groups based on their body weight, with six replicates (cages) per treatment and four rabbits per cage. Rabbits within the same cage were housed together, and each cage served as the experimental unit for all statistical analyses. The four groups were fed experimental diets using PV, PG, MO, and ML as the fiber raw material, respectively. All four fiber raw materials were included at an identical inclusion rate of 25% of the diet on a DM basis. Each fiber ingredient completely replaced the fibrous fraction in the basal formula via DM-based replacement. The four diets were isonitrogenous and isocaloric, with consistent fiber addition levels among all treatments. All experimental diets were formulated into pellets to meet the nutrient requirements of meat rabbits as specified in the Chinese Agricultural Industry Standard (NY/T 4049-2021 [23]). After a 7-day adaptation period, the formal feeding trial lasted for 35 days. All rabbits were fed pelleted diets with daily feed provision, and free access to feed and water was provided throughout the experimental period. The health status of the rabbit herd was monitored daily, and routine indoor disinfection, cleaning, and manure removal were carried out according to standard procedures while maintaining adequate ventilation in the rabbit house. Any sick or dead rabbits were promptly identified, treated appropriately if necessary, and recorded in detail during the entire feeding period.

### 2.7. Sample Collection and Preparation for Animal Trial

At the beginning of the trial, all rabbits in each group were weighed and their initial body weights recorded. Daily feed intake and residual feed amounts were measured on a per-cage (replicate) basis throughout the experiment. The body weight of each rabbit was recorded individually, and the average body weight of the four rabbits within each cage was calculated as the cage mean for subsequent statistical analysis. On the night before the trial concluded (at 20:00), all rabbits were subjected to an overnight fast with free access to water; body weight was then determined at 08:00 the following morning. Total feed consumption over the experimental period was calculated, and the following performance parameters were derived: average daily gain (ADG), average daily feed intake (ADFI), feed-to-gain ratio (F/G), and diarrhea incidence rate. In cases of mortality during the trial, the remaining feed was immediately weighed and relevant data recorded.

On the last day of the trial, all rabbits were subjected to a 12 h fast with ad libitum water supply, and the fasted live weight was defined as the pre-slaughter live weight. Before sampling, all rabbits were weighed, and the individual closest to the cage’s mean weight was chosen. To eliminate sex bias, each treatment group contained an equal number of male and female (1:1 sex ratio). Animals were euthanized via exsanguination by cutting the carotid artery and jugular vein, followed by skin removal for carcass weight measurement. Half-eviscerated carcass weight was obtained after removing the head, trachea, esophagus, all visceral organs and reproductive organs, and eviscerated carcass weight was further measured after eliminating the liver and kidneys. Corresponding slaughter performance indices including dressing percentage, half-eviscerated percentage and eviscerated percentage were calculated based on the above weight data. Longissimus dorsi and quadriceps femoris muscle samples were collected, with surrounding adipose tissue trimmed, for drip loss and shear force detection, respectively. The liver, spleen, thymus and sacculus rotundus were dissected, stripped of attached fat, dried with filter paper and weighed precisely. Immune organ indices were calculated by normalizing each organ weight to the pre-slaughter live weight. Whole blood was collected into pro-coagulant tubes during slaughter, and separated serum was stored for the subsequent detection of serum immune factors. Cecal contents were collected post-slaughter, flash-frozen, and subjected to *16S rRNA* sequencing.

### 2.8. Histological Analysis of Intestinal Morphology

Duodenal and cecal tissue samples were sequentially trimmed, dehydrated, embedded, sectioned, stained, and mounted. After microscopic photography, five fields with intact villi and crypts were selected from each duodenal section, and five fields with clear mucosal layers and intestinal walls were selected from each cecal section for measurement and analysis using Image J software (v1.54f). The villus height and crypt depth in duodenal sections, as well as the mucosal depth and intestinal wall thickness in cecal sections, were quantified, and the average value of the five measurements was calculated as the final result for each sample.

### 2.9. Bioinformatics and Statistical Analysis of Microbiome Data

Total DNA was extracted from fresh feces (~50 mg) using the DNA Stool Mini Kit (51504, QIAGEN, Hilden, Germany). After determining the DNA quality, the region V3 and V4 of *16S rRNA* genes were amplified using the universal primer: forward, 5′-GTGCCAGCMGCCGCGG-TAA-3′; reverse, 5′-GGACTACHVGGGTWTCTAAT-3′. *16S rRNA* gene sequencing analysis was performed by Hangzhou Lianchuan Biotechnology Co., Ltd. (Hangzhou, China) using the Illumina NovaSeq 6000 platform (Illumina Inc., San Diego, CA, USA), targeting the V3–V4 hypervariable regions of the 16S rRNA gene for paired-end sequencing. Briefly, raw paired-end *16S rRNA* gene sequences were demultiplexed and quality-controlled using QIIME 2 (v2023.9). Denoising, chimera removal, and Amplicon Sequence Variant (ASV) inference were performed using the DADA2 plugin with the following parameters: forward reads were truncated at 280 bp and reverse reads at 220 bp, with a maximum expected error threshold of 2.0. To ensure data robustness and exclude sequencing noise, only ASVs with a total relative abundance ≥ 0.005% across all samples and present in at least 20% of the samples within any experimental group were retained for downstream analysis. The average sequencing depth per sample was approximately 39,250 ± 7213 (mean ± SD) valid reads, with Q30 scores exceeding 92%. Taxonomic assignment of ASVs was performed using the Silva database (v138) against representative sequences. Alpha diversity indices (Chao1, observed species and Ace) were calculated to evaluate community richness and evenness, with differences among groups assessed via the Wilcoxon rank-sum test. Beta diversity was visualized using principal coordinate analysis (PCoA) based on UniFrac distances, and statistical differences in bacterial community structure among groups were evaluated using ANOSIM. For differential abundance analysis, to detect overall group effects prior to pairwise comparisons, we first performed Kruskal–Walli’s rank-sum test to screen for globally significant taxa. Subsequently, for all pairwise post hoc multiple comparisons between specific treatment groups, we applied the two-stage linear step-up procedure of Benjamini, Krieger, and Yekutieli (BKY) to strictly control the false discovery rate (FDR). This two-stage adaptive procedure offers enhanced FDR control under the dependency structures commonly observed in sparse microbiome data. Taxa with an adjusted q-value < 0.05 were considered significantly differentially abundant between groups. It is important to note that while Linear Discriminant Analysis Effect Size (LEfSe) was employed as an exploratory visualization tool (with LDA score > 2) to illustrate the most discriminant biomarkers, the definitive statistical significance of differential taxa was rigorously determined exclusively by the FDR-corrected BKY post hoc tests, ensuring the robustness and reproducibility of our conclusions [24]. To predict the functional potential of the cecal microbial communities, the PICRUSt2 software package (version pícrust2.2.0b) was employed. Raw sequencing reads have been deposited in the Genome Sequence Archive of the National Genomics Data Center under accession number CRA027667.

### 2.10. Serum Physiology and Biochemistry Index Analysis

Serum glucose, triglycerides, total cholesterol, high-density lipoprotein cholesterol, low-density lipoprotein cholesterol, free fatty acids, and blood urea nitrogen were determined using an automatic biochemical analyzer (Mindray, Shenzhen, China) with commercial enzymatic kits (Nanjing Jiancheng Bioengineering Institute, Nanjing, China). Serum malondialdehyde and total antioxidant capacity were measured using commercial colorimetric kits (TBA method for malondialdehyde, FRAP method for total antioxidant capacity) on a microplate reader (Rayto Life Science Co., Ltd., Shenzhen, China) [25,26]. Serum tumor necrosis factor-α, D-lactate, diamine oxidase, lipopolysaccharide, and lipopolysaccharide-binding protein were quantified using rabbit-specific ELISA kits (Meilian Biotechnology Co., Ltd., Shanghai, China), strictly following the manufacturers’ protocols [27]. Absorbance was read at 450 nm, and concentrations were calculated from standard curves. All assays were performed in triplicate.

### 2.11. Statistical Analysis

All experimental data were preliminarily organized using Microsoft Excel software (Microsoft Corporation, Redmond, WA, USA), and statistical analyses were performed using SPSS 27.0 software (IBM Corp., Armonk, NY, USA). The cage (four rabbits per cage) was considered as the experimental unit for all statistical analyses. For growth performance parameters (ADG, ADFI, and F/G), individual rabbit body weight data were first averaged to obtain a single value per cage, and feed intake was recorded on a per-cage basis; these cage-level values were then used for statistical analysis. Normality (Shapiro–Wilk test) and homogeneity of variances (Levene’s test) were first assessed for all quantitative variables. For data that followed a normal distribution and had homogeneous variances, one-way analysis of variance (ANOVA) was performed; when the ANOVA result was significant (*p* < 0.05), Duncan’s multiple range test was used for post hoc comparisons. For data with heterogeneous variances, Welch’s ANOVA was conducted followed by Games–Howell post hoc test. Outliers were identified using the box-plot method, and values that were obviously erroneous or exceeded ± 3 standard deviations from the mean were excluded, while the remaining values were retained. Diarrhea incidence was analyzed using a binomial generalized linear model with logit link and cage as the experimental unit. The overall treatment effect was evaluated by a likelihood-ratio χ^2^ test, and pairwise comparisons were conducted using Fisher’s protected least significant difference test. For differential abundance of the microbiome, the two-stage linear step-up procedure of Benjamini, Krieger, and Yekutieli was used for post hoc multiple comparisons to control the FDR. Unless otherwise specified, results were expressed as mean ± standard error of the mean (SEM). *p* < 0.01 was considered extremely significant, *p* < 0.05 was considered statistically significant, and a *p* value between 0.05 and 0.10 was considered a tendency towards a difference. Significant differences in tables were indicated using the letter marking method, where different lowercase letters indicated significant differences between groups (*p* < 0.05), and the same lowercase letter indicated no significant difference between groups (*p* > 0.05).

## 3. Results

### 3.1. Basic Properties of Fiber Raw Materials from Different Sources

Table 1 summarizes the proximate analysis of the 12 experimental fiber raw materials. All samples exhibited high DM content ranging from 85.55% to 98.34%, which favors long-term storage. Substantial differences in CP contents were observed among the tested materials. MO contained the highest CP (10.37%), whereas bamboo fiber had the lowest CP (0.13%). Leaf-type forage resources (MO, PV, ML) showed relatively greater CP concentrations compared to crop straws, colloidal fiber and purified fiber products. Marked variations were also detected in CF content. Bamboo fiber presented the highest CF (57.21%), followed by konjac flour (45.78%). Paper mulberry leaf meal had the lowest CF (15.65%), while CF was undetectable in sodium alginate. In terms of fiber fractions, bamboo fiber had the greatest NDF (81.06%) and ADF (58.12%) across all materials. Leaf forages (PV, MO, ML) exhibited relatively low NDF and ADF values. NDF and ADF could not be quantified for sodium alginate owing to its unique colloidal structure.

### 3.2. In Vitro Hydration Properties and Fermentation Characteristics

**Figure 1:**
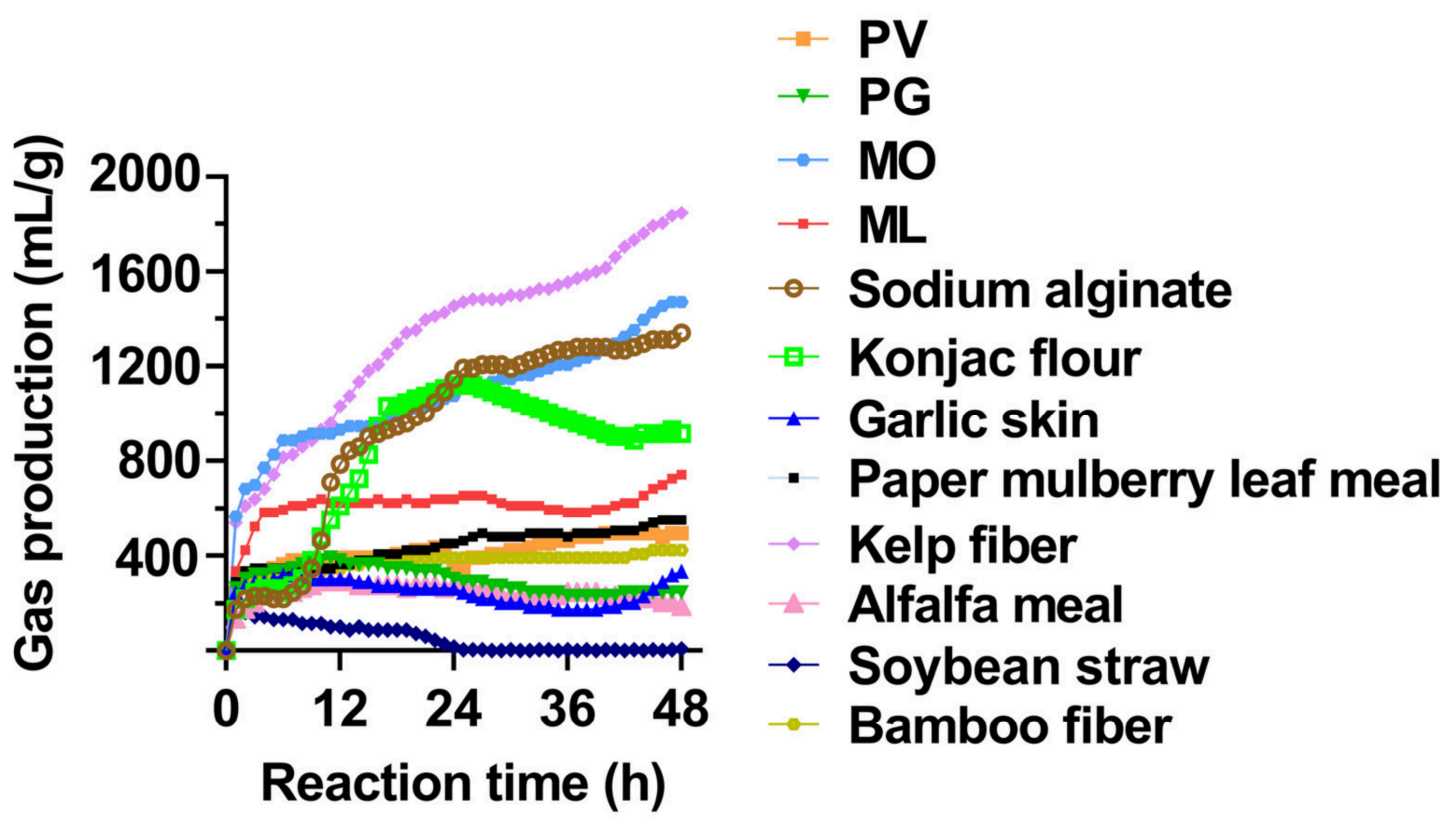
In vitro gas production curves of fibers with different physicochemical properties. Gas production was recorded continuously at 2 h intervals throughout the incubation. Each curve represents the mean gas production profile of a single fiber source. PV, peanut vine; PG, *Pennisetum giganteum* meal; MO, *Moringa oleifera* leaf meal; ML, mulberry leaf meal.

Table 2 shows that the 12 fiber raw materials varied greatly in in vitro WBC, WSC, and viscosity. In terms of WBC, konjac flour exhibited the maximum value, while bamboo fiber had the lowest WBC. For WSC, konjac flour and sodium alginate were markedly higher than all other fiber materials. When it came to viscosity, konjac flour and sodium alginate showed far higher viscosity values, whereas garlic skin possessed the minimum viscosity. Numerically, colloidal-derived fiber raw materials (konjac flour, sodium alginate) possessed the strongest overall hydration properties. Table 2 and Figure 1 present the 48 h gas production kinetic parameters, dynamic cumulative gas production curves and VFA concentrations of 12 fiber raw materials with distinct sources. Gas production was recorded continuously every 2 h during the entire incubation period, and each curve in Figure 1 represented the average gas production change rule of a single fiber material. Kelp fiber had the maximum cumulative gas yield at the end of fermentation, followed by sodium alginate and konjac flour. The gas production of kelp fiber maintained a sustained rapid upward trend within 48 h. Sodium alginate and konjac flour rose rapidly in the early fermentation stage, while their gas accumulation rate slowed down after 24 h of incubation. In contrast, soybean straw and bamboo fiber possessed the lowest total gas output; soybean straw even showed a slight drop in gas production after 30 h, indicating insufficient fermentable substrates. ML and MO exhibited moderate gas production rate and total gas volume. Notably, MO had a sharp rise in gas yield at the early stage, which was consistent with its highest IR_0_ in Table 2. Garlic skin, alfalfa meal and paper mulberry leaf meal maintained slow and stable gas accumulation throughout the whole fermentation process. Kelp fiber, sodium alginate and konjac flour produced markedly higher acetate and total VFA concentrations than other feedstuffs, revealing their superior fermentation capacity. In this study, we did not directly measure the extent of degradation or disappearance of substrate dry matter, but instead concentrated the evaluation on the end products of microbial metabolism. Given that VFAs are direct products of fiber degradation, and their production yields have been demonstrated to more accurately reflect the actual utilization efficiency of substrates by hindgut microbiota than gas production kinetic parameters [28,29], we selected accumulated VFA production as the core quantitative indicator for comparing the fermentation performance among different fiber raw materials.

We analyzed the Spearman correlation coefficients between in vitro hydration properties and fermentation characteristics of 12 fibrous raw materials. To further explore whether these associations were independent of fiber composition, we also performed partial correlation analysis controlling for dry matter, crude protein, and major fiber fractions (CF, NDF and ADF) (Table 3).

In the zero-order correlation analysis, for gas production indices, viscosity was significantly positively correlated with GP48 (r = 0.58; *p* < 0.05). WBC, WSC and viscosity were all positively correlated with K, and WSC showed an extremely significant correlation with K (r = 0.83; *p* < 0.01). No significant correlations were found between the three hydration indicators and IR_0_ × 100 or T½. In addition, WBC and WSC had no obvious correlation with NH_3_-N, while viscosity was non-significantly negatively correlated with NH_3_-N (r = −0.55; *p* > 0.05). For volatile fatty acids, WSC was significantly positively correlated with acetate, propionate, total SCFAs and total VFAs (r = 0.68, 0.61, 0.67, 0.61, respectively; *p* < 0.05), and exhibited an extremely significant positive correlation with SCFAs/VFAs (r = 0.75; *p* < 0.01). Only WBC had a significant positive correlation with valerate (r = 0.66; *p* < 0.05). Viscosity presented extremely significant negative correlations with isobutyrate, isovalerate and total BCFAs (r = −0.84, −0.84, −0.87, respectively; *p* < 0.01), whereas WBC and WSC showed no significant associations with all BCFA-related indicators. No significant correlations between the three hydration properties and butyrate were observed (*p* > 0.05).

In the partial correlation analysis after controlling for DM, CP and fiber composition, several previously significant associations were attenuated, whereas certain relationships involving fermentation kinetics and butyrate remained significant. Specifically, WBC exhibited a significant negative partial correlation with IR_0_ × 100 (r = −0.84, *p* < 0.05), and viscosity also showed a significant negative partial correlation with IR_0_ × 100 (r = −0.77, *p* < 0.05). Both WBC and WSC remained significantly positively correlated with K (r = 0.75 and 0.89, respectively; *p* < 0.05), and viscosity showed an extremely significant positive partial correlation with K (r = 0.97, *p* < 0.05). Notably, WSC and viscosity both exhibited significant positive partial correlations with butyrate (r = 0.68 and 0.71, respectively; *p* < 0.05), a relationship that was not evident in the zero-order analysis. WSC also remained significantly positively correlated with total SCFAs (r = 0.66, *p* < 0.05). However, after controlling for fiber composition, the previously significant correlations between WSC and acetate, propionate, total VFAs, and SCFAs/VFAs were no longer significant (*p* > 0.05). Similarly, the significant negative correlations between viscosity and BCFAs observed in the zero-order analysis disappeared after partial correlation adjustment. Collectively, hydration properties, especially WSC and viscosity, were associated with fermentation parameters even after controlling for DM, CP and fiber composition.

### 3.3. Principal Component Analysis (PCA) and Cluster Analysis

**Figure 2:**
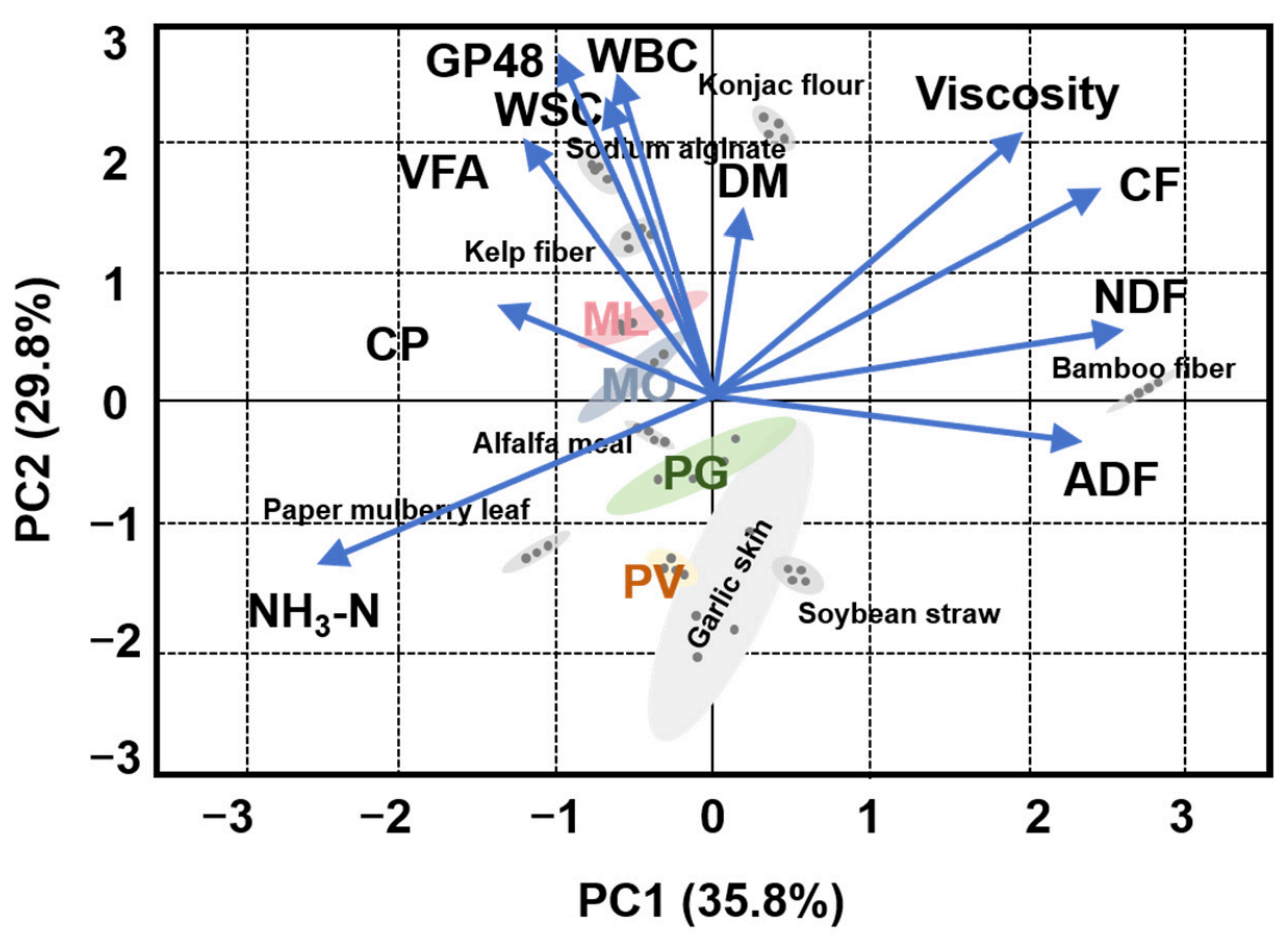
Biplot of PCA based on nutritional, hydration and fermentation characteristics of 12 fibers feed ingredients. Blue vectors represent the loading of each measurement index, and scattered points correspond to individual fiber raw material samples. Peanut vine meal (PV), Pennisetum giganteum meal (PG), moringa leaf meal (MO) and mulberry leaf meal (ML) were distributed along the PC1 axis (corresponding to NDF, ADF and CF) and far away from the viscosity vector, indicating that the four samples had similar fiber composition characteristics and low viscosity levels. PV, PG, MO and ML showed a gradient distribution along the PC2 axis (corresponding to WBC, WSC, GP48h and VFA), reflecting a continuous gradient variation in water-absorbing capacity and in vitro fermentation activity among the samples.

To further systematically distinguish the comprehensive physicochemical, nutritional and fermentation differences among the 12 fibrous raw materials, PCA was conducted based on 11 relevant indices (Table 4 and Figure 2). Three principal components with eigenvalues > 1.0 were extracted, together accounting for 80.7% of the total variance, thereby capturing the majority of information in the original dataset. PC1 explained 35.8% of the variance and was primarily defined by fiber fractions (NDF, ADF, CF) and viscosity. PC2 accounted for 29.8% of the variance and was mainly associated with WBC, WSC, GP48h and VFA. PC3 contributed 15.1% of the variance, with CP, DM, and NH_3_-N as its core loading variables. Collectively, PC1 and PC2 jointly explained 65.6% of the total variation, indicating that hydration properties and fiber fractions were the primary differentiating factors, followed by fermentation characteristics, while conventional nutritional indices exerted the weakest discriminatory power. Subsequently, k-means clustering was performed based on the principal component scores of the 12 fiber raw materials (Table 5). Cluster I only contained PV. Cluster II included PG, bamboo fiber and soybean straw. Cluster III comprised MO and alfalfa meal. Cluster IV included ML, garlic skin, paper mulberry leaf meal, kelp fiber, konjac flour and sodium alginate.

After determining the nutritional properties, in vitro hydration properties, and fermentation characteristics of the 12 fiber raw materials, we screened the candidates for subsequent animal trials according to the following criteria: (1) Konjac flour and sodium alginate were excluded because of their excessively high viscosity, which tends to form a physical barrier in the intestine, hindering the thorough mixing of digesta with digestive enzymes and intestinal transit, and may impose a considerable burden on intestinal digestion in rabbits under ad libitum feeding. (2) Bamboo fiber was excluded because its NDF and ADF are as high as 81.06% and 58.12%, respectively, which are extreme outliers; if added at the same inclusion rate as the other fibers, it would severely unbalance the dietary fiber structure and compromise the basic comparability among groups. (3) Although kelp fiber shows high gas production, it was not suitable for direct comparison given that its chemical composition may differ substantially from that of terrestrial plant fibers, and it also has relatively high viscosity and high cost. (4) The hydration–fermentation profiles of PG, garlic skin, alfalfa meal, and paper mulberry leaf meal partially overlap or are similar; including all of them in the in vivo trial would increase the number of treatments without providing new differentiating dimensions. (5) PV is widely used as a fiber source for meat rabbits in South China and can serve as a regional baseline reference for this experiment [16]. (6) Soybean straw had the lowest GP48 and total SCFAs, showing little fermentative activity, and its low fermentation overlapped with that of PV. Based on the above exclusion criteria, and considering the limited experimental scale, the four fibers PV, PG, MO, and ML were selected. They have relatively low and comparable viscosities, their fiber fractions are within the utilizable range for meat rabbits, and their hydration properties and fermentation indices generally show an increasing trend from low to high, adequately covering the typical spectrum of hydration–fermentation combinations (Table 5). Therefore, they were chosen for the in vivo trial to verify the differential effects of fibers with distinct hydration properties and fermentation on the intestinal health and growth performance of meat rabbits (Table 6).

### 3.4. Growth Performance

The growth performance parameters of the test rabbits are shown in Table 7. The initial body weight did not differ significantly among all experimental groups before the trial (*p* > 0.05), indicating consistent initial growth conditions for all animals. After the feeding trial, significant differences were observed in final body weight, ADFI, ADG, and diarrhea rate across groups (*p* < 0.05), whereas no significant differences were detected in F/G among treatments (*p* > 0.05). Notably, the PV, PG and MO groups had similar ADFI values that were markedly higher than the ML group. The GLM analysis revealed a significant overall effect of fiber type on diarrhea incidence (*p* < 0.05). Pairwise comparisons using Fisher’s protected LSD further indicated that the ML group had a significantly higher diarrhea rate than the MO group (*p* < 0.05), while no other differences were detected (*p* > 0.05).

### 3.5. Carcass Performance and Organ Development

Table 8 presents the impacts of four fiber raw materials with distinct physicochemical characteristics on the visceral organ weight, organ indices, carcass weight and dressing percentage of New Zealand white rabbits. Significant intergroup differences were observed only in appendix weight, half-eviscerated carcass weight, and eviscerated carcass weight (*p* < 0.05), whereas no significant differences were detected among groups in the absolute weights of the lung, heart, liver, spleen, kidney, round sac, appendix, or any of the organ indices (*p* > 0.05). Notably, the ML group consistently exhibited the lowest values for appendix weight, half-eviscerated carcass weight, and eviscerated carcass weight among all treatments.

### 3.6. Meat Quality

**Figure 3:**
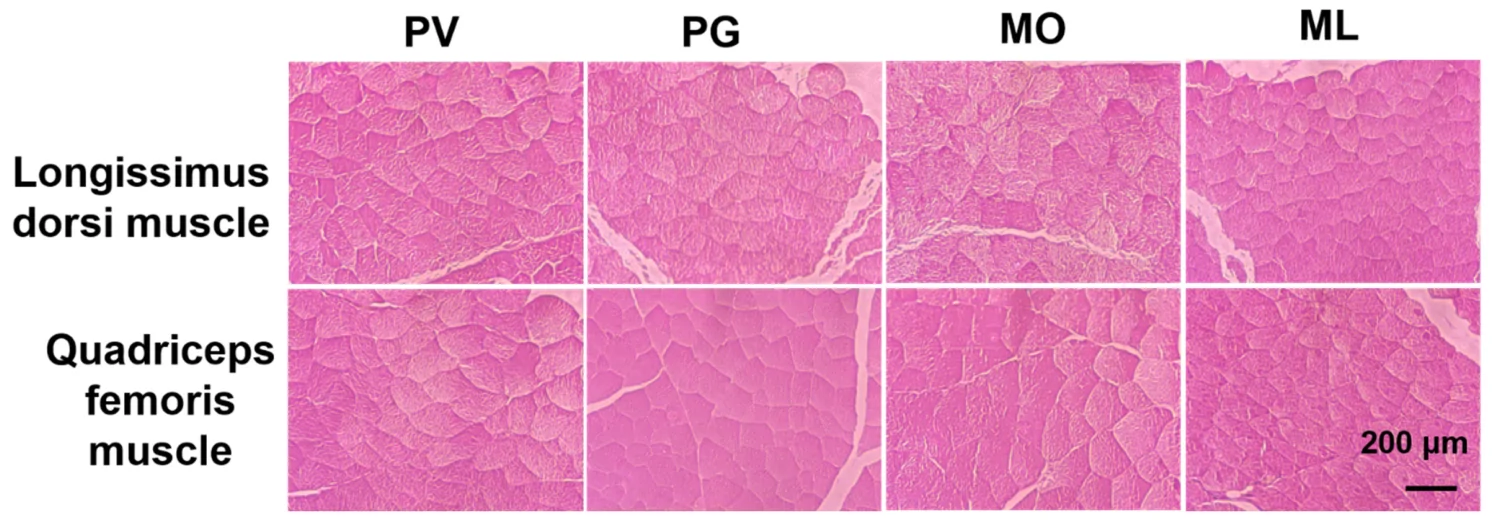
H&E-stained cross-sections of longissimus dorsi muscle and quadriceps femoris muscle from New Zealand white rabbits fed diets containing different fiber raw materials. Groups: PV, PG, MO, and ML represent rabbits fed diets containing different fiber raw materials. Scale bar = 200 μm. No obvious pathological changes were observed in muscle fibers across all groups. PV, peanut vine; PG, *Pennisetum giganteum* meal; MO, *Moringa oleifera* leaf meal; ML, mulberry leaf meal.

The effects of the four fiber raw materials (PV, PG, MO, and ML) with contrasting physicochemical properties on meat quality traits of New Zealand white rabbits are presented in Table 9 and Figure 3. In the longissimus dorsi muscle, drip loss, shear force, and single muscle fiber cross-sectional area did not differ significantly among treatments (*p* > 0.05). Likewise, no significant treatment effects were observed for drip loss or the muscle fiber cross-sectional area of the quadriceps femoris (*p* > 0.05). However, the shear force of the quadriceps femoris showed a significant difference among groups (*p* < 0.05), with the MO group exhibiting the lowest value, significantly lower than that of the PV and ML groups.

### 3.7. Serum Physiology and Biochemistry

Four types of dietary fiber with distinct physicochemical properties exerted no significant intergroup effects on serum glycolipid metabolism, nitrogen metabolism and systemic antioxidant-related indices of New Zealand white rabbits (Table 10; *p* > 0.05). However, significant treatment effects were observed for indicators associated with intestinal inflammation and intestinal barrier function. Specifically, serum tumor necrosis factor-α, D-lactate, diamine oxidase and lipopolysaccharide differed significantly among groups (*p* < 0.05), whereas lipopolysaccharide-binding protein showed no significant intergroup differences (*p* > 0.05). Notably, the serum concentrations of tumor necrosis factor-α, D-lactate and diamine oxidase in the ML group were markedly higher than those in the PV, PG and MO groups (*p* < 0.05).

### 3.8. Duodenal and Cecal Intestinal Morphology

**Figure 4:**
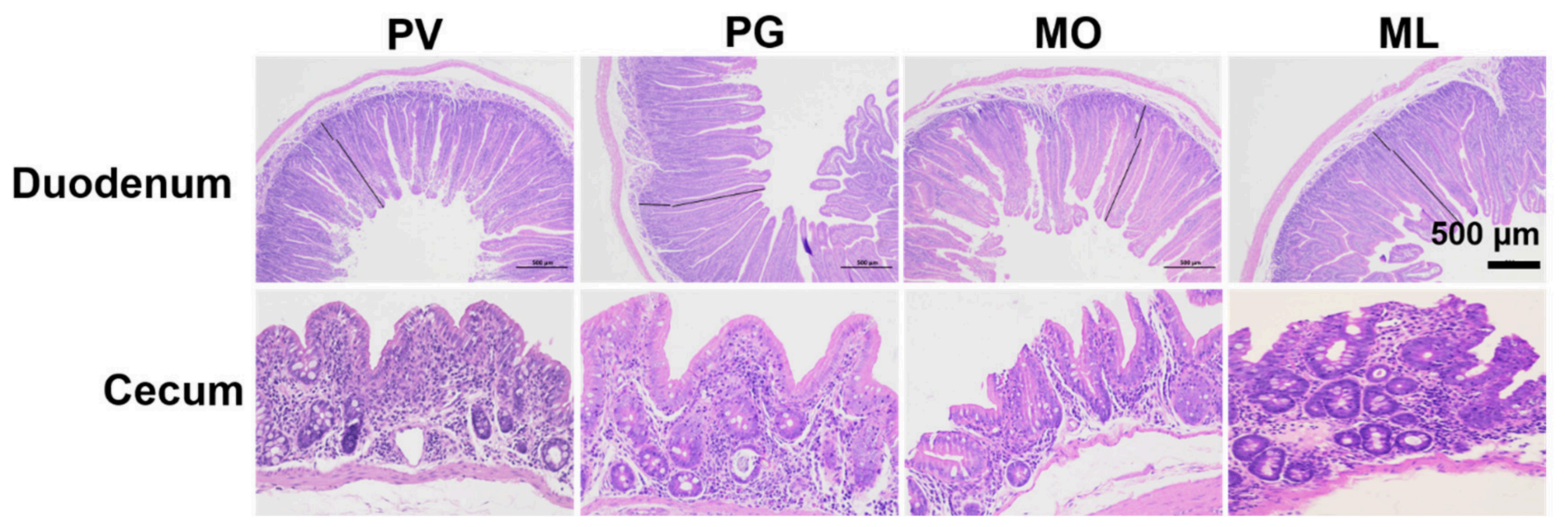
H&E-stained sections showing the effects of dietary fiber with different physicochemical properties on duodenal and cecal morphology in New Zealand white rabbits. Groups: PV, PG, MO, and ML represent rabbits fed diets containing different fiber raw materials. The lines in the duodenal images indicate villus height and crypt depth; those in the cecal images indicate mucosal depth and intestinal wall thickness. The scale bar in the figure represents 500 μm. PV, peanut vine meal; PG, *Pennisetum giganteum* meal; MO, *Moringa oleifera* leaf meal; ML, mulberry leaf meal.

The effects of dietary fibers with distinct physicochemical properties on the intestinal morphology of the duodenum and cecum in New Zealand white rabbits are presented in Table 11 and Figure 4. No significant intergroup differences were observed in duodenal morphological parameters across all treatments (*p* > 0.05). In contrast, significant treatment effects were detected for cecal mucosal depth (*p* < 0.05) and cecal wall thickness (*p* < 0.05). Notably, the ML group exhibited markedly greater cecal mucosal depth and intestinal wall thickness compared to all other groups. This finding suggests that dietary supplementation with ML may exert adverse impacts on cecal tissue development in meat rabbits.

### 3.9. Cecal Microbiota and VFAs

**Figure 5:**
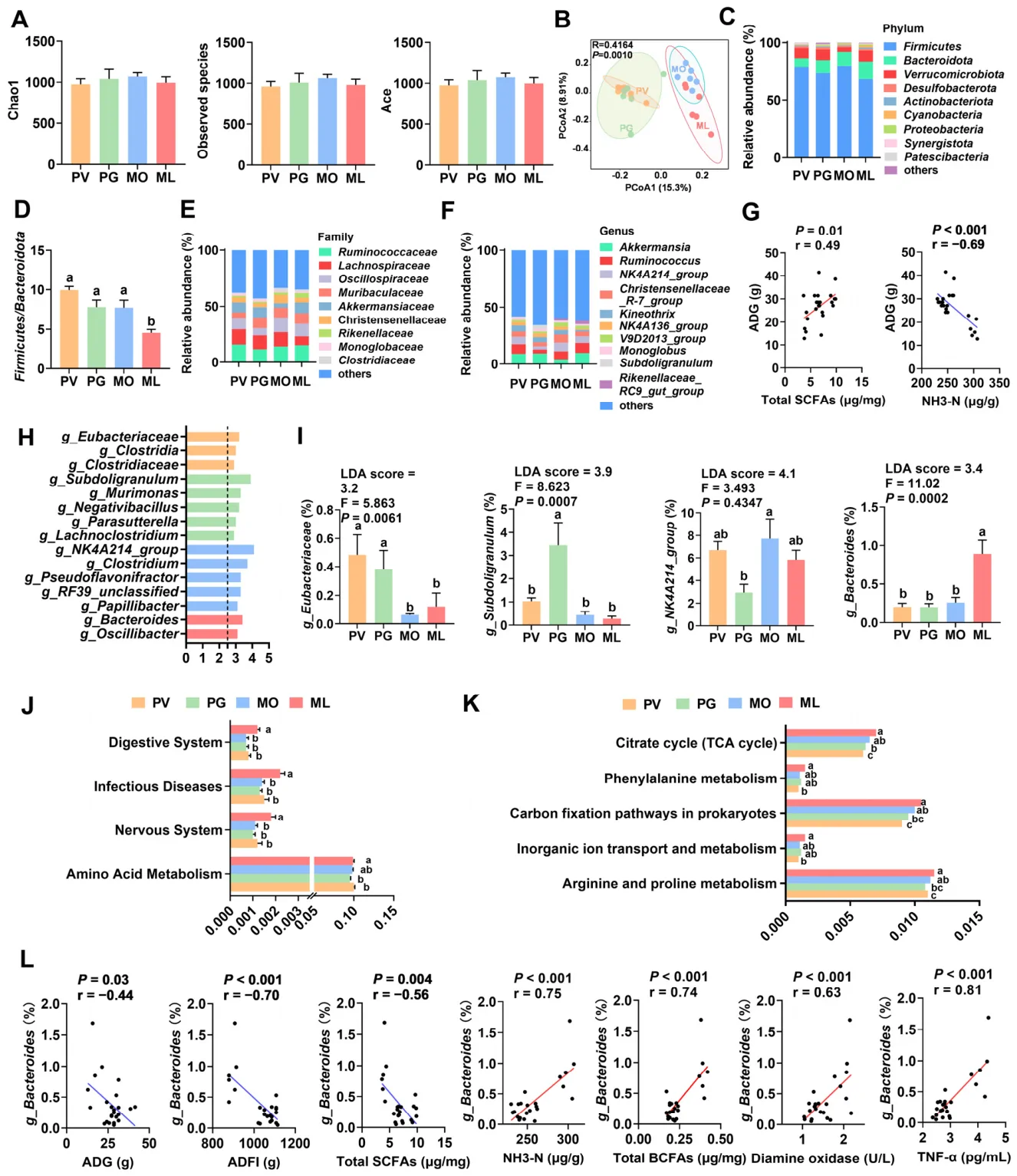
Effects of dietary fiber with different physicochemical properties on intestinal microbiota of rabbits. (**A**) Alpha diversity indices (Chao1, observed species and Ace). (**B**) Principal coordinate analysis (PCoA) of cecal bacterial communities. The *p* value was calculated by ANOSIM analysis. (**C**) Taxonomic composition of bacteria at the phylum level. (**D**) The ratio of Firmicutes to Bacteroidetes. (**E**,**F**) Stacked bar charts of bacterial relative abundance at the family and genus levels. (**G**) Simple linear regression analysis illustrating correlations between ADG and SCFAs and NH_3_-N. (**H**) LEfSe analysis showing differentially enriched genera among groups. (**I**) Relative abundance of representative biomarker genera. *p* values were adjusted using the two-stage linear step-up procedure of Benjamini, Krieger, and Yekutieli for multiple comparisons. (**J**,**K**) Predicted KEGG functional pathways of cecal microbiota. (**L**) Simple linear regression analysis illustrating correlations between the relative abundance of Bacteroides and growth performance, fermentation metabolites and inflammatory indicators. PV, peanut vine meal; PG, *Pennisetum giganteum* meal; MO, *Moringa oleifera* leaf meal; ML, mulberry leaf meal. All values are presented as means ± standard error of the mean (SEM). ^a–c^ Means with different superscripts within a row differ significantly (*p* < 0.05).

The alpha diversity results showed that Chao1, observed species and Ace indices exhibited no intergroup differences (Figure 5A; *p* > 0.05). PCoA analysis revealed the distinct separation of cecal microbial communities among four treatments (Figure 5B; R = 0.4164, *p* < 0.001), indicating dietary fiber with different physicochemical properties reshaped gut microbiota structure drastically. Stacked bar charts at phylum, family and genus levels further illustrated taxonomic distribution differences (Figure 5C–F). At the phylum level, *Firmicutes* and *Bacteroidota* dominated the cecal microbiota of rabbits. The MO group had the highest relative abundance of *Firmicutes*, whereas ML significantly increased the proportion of *Bacteroidota* and reduced the *Firmicutes*/*Bacteroidota* ratio. At the family level, SCFA-producing *Lachnospiraceae* and *Ruminococcaceae* were more abundant in the MO group. At the genus level, intestinal barrier-protective taxa including *Akkermansia* and *Christensenellaceae_R-7_group* were remarkably enriched in MO rabbits, while the pro-inflammatory, protein-degrading genus *Bacteroides* occupied a higher proportion in the ML group. Consistent with microbial composition, cecal fermentation metabolites exhibited obvious divergence among treatments (Table 12). The MO group possessed the highest concentrations of acetate, propionate, butyrate and total SCFAs, together with the lowest levels of BCFAs. By contrast, the ML group showed markedly reduced total SCFA concentration, elevated BCFA accumulation and the highest NH_3_-N content. Simple linear regression analysis (Figure 5G) showed that ADG was positively correlated with total SCFA concentration (r = 0.49, *p* < 0.05), whereas ADG was negatively correlated with cecal NH_3_-N concentration (r = −0.69, *p* < 0.001). Furthermore, LEfSe analysis was conducted to screen biomarker genera specific to each group (Figure 5H; LDA > 2.5, *p* < 0.05). The characteristic taxon of the PV group belonged to *Eubacteriaceae*; the PG group was enriched with the polysaccharide-degrading genus *Subdoligranulum*; the MO group had an overabundance of the beneficial fiber-degrading taxon *NK4A214_group*; and the biomarker genus of the ML group was the protein-degrading *Bacteroides* (Figure 5I). KEGG functional prediction suggested that microbial pathways related to protein catabolism and inflammatory stress, including amino acid metabolism, the TCA cycle, and arginine and proline metabolism, were significantly enriched in the ML group (Figure 5J). The MO group exhibited higher expression of prokaryotic carbon fixation pathways associated with carbohydrate utilization (Figure 5K). Simple linear correlation analysis was performed to quantify the relationships between the relative abundance of Bacteroides and phenotypic indicators (Figure 5L). The relative abundance of Bacteroides exhibited significant negative correlations with ADG (r = −0.44, *p* = 0.03), ADFI (r = −0.70, *p* < 0.001) and total SCFA concentration (r = −0.56, *p* = 0.004). Meanwhile, *Bacteroides* abundance was positively and significantly correlated with cecal NH_3_-N concentration (r = 0.75, *p* < 0.001), serum diamine oxidase activity (r = 0.63, *p* < 0.001) and serum tumor necrosis factor-α concentration (r = 0.81, *p* < 0.001). Collectively, these microbial and fermentation metabolite data demonstrated that MO was associated with increased cecal microbial diversity, the enrichment of SCFA-producing bacteria, and enhanced SCFA production. By contrast, ML was associated with gut dysbiosis and the specific enrichment of Bacteroides, which were correlated with reduced SCFA production, elevated BCFAs and NH_3_-N concentrations, and markers of intestinal inflammation.

## 4. Discussion

This study combined in vitro physicochemical characterization and in vivo feeding trials to investigate the effects of four dietary fibers (PV, PG, MO, and ML) with similar low viscosity but distinct hydration and fermentation gradients on intestinal health and growth performance of meat rabbits. Among them, PV served as the regional baseline reference, as it is widely used as a fiber source in meat rabbit diets in South China. Moreover, the hydration properties and fermentation indices of PV, PG, MO, and ML generally showed an increasing trend from low to high, covering different hydration–fermentation combinations. The core findings of this study showed that MO was associated with optimized cecal microbiota, increased SCFA production, and lower diarrhea incidence; by contrast, despite its high in vitro fermentation, ML was associated with intestinal inflammation, reduced SCFAs, ADG, and ADFI, as well as elevated BCFAs, ammonia nitrogen, and diarrhea rate in vivo. This contradiction does not negate the value of in vitro methods, but rather reveals that in vitro fermentation parameters reflect the potential fermentation of substrates under idealized homogenized conditions, and cannot be directly extrapolated to actual physiological effects in vivo [6]. Therefore, we emphasize that when evaluating the nutritional value of novel fiber raw materials, in vitro predictions must be combined with in vivo validation, and all conclusions should be strictly confined to the fiber types, animal models, and feeding conditions used in this trial, avoiding extrapolation of in vitro data or the present results to untested fibers or species.

The proximate and fiber fraction data provided a static picture of chemical composition, but their biological relevance only became clear when interpreted alongside hydration and fermentation indices. For example, bamboo fiber and konjac flour both contained exceptionally high CF levels (57.21% and 45.78%, respectively), yet their hydration behaviors and fermentabilities were diametrically opposed. Bamboo fiber, with its rigid lignocellulosic matrix, exhibited minimal WBC and negligible fermentation. Konjac flour, despite its high CF value, is rich in glucomannan and formed a highly viscous gel that was intensely fermentable in vitro [8]. This contrast highlights a key limitation of relying on CF or NDF alone to predict physiological function: the physical form and water-interaction properties of fiber can override compositional descriptors. It is worth emphasizing that the findings of this study do not negate the association between in vitro hydration properties and fermentation. Zero-order Spearman correlation analysis revealed that viscosity was negatively correlated with BCFA production, while WSC was positively correlated with acetate, propionate, and total SCFAs, suggesting that fibers with higher water solubility may be more readily degraded by microorganisms in vitro. This result is consistent with our previous study in weaned piglets, which also demonstrated a positive correlation between in vitro hydration properties and SCFA production [8]. However, partial correlation analysis controlling for fiber composition revealed that the WSC–acetate/propionate associations were largely confounded by fiber matrix, whereas WSC remained correlated with butyrate and total SCFAs, and viscosity remained correlated with butyrate and fermentation kinetic parameters (K and IR_0_). These findings suggest that certain hydration-related effects on fermentation, particularly those involving butyrogenic potential and fermentation dynamics, are genuine rather than compositional artifacts. Nevertheless, such correlations should be interpreted with caution, as causal verification requires further studies using purified fibers or modified fiber.

An unexpected finding of this study was that ML and MO exhibited markedly different fermentation fates and physiological effects once introduced into the rabbit cecum. Although both fibers showed high cumulative gas production and total SCFA concentrations in vitro, ML-fed rabbits displayed an enrichment of *Bacteroides* [30], a genus known for its protein-degrading capacity, along with elevated BCFAs and NH_3_-N concentrations [7,31], and reduced SCFAs in vivo. A possible explanation is that the strong water-holding capacity of ML may cause excessive swelling and the formation of a gel-like matrix in the digestive tract, which could limit microbial access to structural polysaccharides and shift microbial metabolism toward the utilization of endogenous proteins and residual dietary peptides as alternative substrates [7,9,32]. In contrast, the MO group maintained a saccharolytic community dominated by *Lachnospiraceae*, *Ruminococcus*, and the *NK4A214_group*, which are known producers of acetate, propionate, and butyrate [33,34]. It should be emphasized, however, that whether the excessive hydration properties of ML and the associated gut dysbiosis are directly responsible for the impaired intestinal function and growth performance in the ML group remains to be confirmed by further studies using purified fibers in combination with fecal microbiota transplantation. Moreover, the progression from microbial dysbiosis to intestinal barrier breakdown was evident in the serological profile of ML-fed animals. The simultaneous elevation in tumor necrosis factor-α, D-lactate, diamine oxidase, and lipopolysaccharide points toward increased intestinal permeability and endotoxin translocation, a classic signature of a disrupted gut barrier [35]. The morphological changes in the cecum, marked by greater mucosal depth and wall thickness, are consistent with a compensatory hyperplastic response to chronic low-grade inflammation rather than healthy tissue development. Notably, the correlation analysis positioned Bacteroides abundance as a central node in this pathology, with strong positive associations with serum tumor necrosis factor-α, diamine oxidase activity, and cecal ammonia concentration, and negative correlations with growth performance. Collectively, these findings raise the hypothesis that excessive fiber hydration may influence microbial accessibility and potentially promote proteolytic dysbiosis, which could contribute to barrier damage and systemic inflammation. However, the underlying relationships and mechanisms need further validation.

Beyond the effects on gut health, MO feeding was associated with improved meat tenderness, as reflected by the significantly lower shear force of the quadriceps femoris in the MO group compared to the PV and ML groups. Shear force is a direct indicator of meat tenderness, with lower values indicating greater tenderness [36]. This observation in the MO group is consistent with a previous report on Moringa oleifera leaf supplementation in rabbits [37], but it is also notable that in the present study the tenderness improvement was specific to the quadriceps femoris and was not detected in the longissimus dorsi. Such muscle-specific responses have been documented in other nutritional intervention studies (e.g., with vitamin E) [38], though the comparability of those studies with our fiber-based regimen remains unknown. In our trial, the MO group showed a saccharolytic microbial community enriched in SCFA-producing genera, along with a higher antioxidant capacity. Some earlier studies have associated similar microbial or antioxidant changes with meat quality traits in other animal models [16], but direct evidence connecting cecal microbiota composition to postmortem tenderization in rabbits is still limited. In summary, the present study shows that MO feeding is correlated with lower shear force specifically in the quadriceps femoris, but no causal link can be inferred from these data, and further experiments are required to clarify the physiological basis of this site-specific response.

Several limitations should be acknowledged. (i) The mechanism underlying the in vitro–in vivo fermentation discrepancy of ML was not verified. We did not measure digesta viscosity or passage rate in vivo, and therefore the proposed physical mechanism could not be validated [9,32]. In addition, because we did not determine the contents of minerals, bioactive compounds, or potential anti-nutritional factors, we were also unable to verify whether these components contributed to the observed discrepancy. These aspects will be considered in future studies. (ii) The four fiber raw materials (PV, PG, MO, and ML) were naturally occurring materials in which hydration properties and fermentation covaried, so this design does not allow the independent assessment of their individual effects. Furthermore, although the diets were isonitrogenous and isocaloric with equal fiber inclusion, they differed in NDF, ADF, ADL, and SDF contents, meaning that the actual supply of fermentable carbohydrates was not strictly equivalent across treatments. These confounding factors may have collectively influenced the observed outcomes. Future studies using purified or chemically modified fibers with independently manipulated hydration and fermentation are needed to dissect their respective contributions. (iii) Owing to constraints in the scale of the animal experiment, including the number of treatment groups, animal availability, and housing capacity, the in vivo study selected only four fiber types with distinct hydration and fermentation characteristics, which did not cover all possible fiber types. Consequently, the gradient descriptions in this study are based on the order of measured values, rather than on statistically confirmed thresholds. It should be noted that we did not directly measure the disappearance of substrate dry matter, but instead focused on microbial metabolites. Given that VFAs are direct products of fiber degradation and their yields are superior to gas production parameters in reflecting hindgut utilization efficiency [28,29], we used accumulated VFA production as the core indicator for comparing fermentation performance across fiber raw materials. (iv) Strictly speaking, this in vivo feeding trial lacked a universally recognized standard control group. Nevertheless, we adopted PV as the regional baseline reference fiber source, as it is the dominant and widely used fibrous feedstuff in commercial meat rabbit production in Fujian Province and South China. All experimental diets were formulated to be isonitrogenous, isocaloric, and with identical dietary fiber addition levels, allowing us to compare the three alternative fiber raw materials against this practical local baseline. (v) Metagenomic sequencing and qPCR of functional genes were not performed in this study, which limits our ability to conduct in-depth functional profiling of the cecal microbiota beyond PICRUSt2-based predictions. Consequently, we cannot provide definitive evidence at the genetic level for the inferred shift from carbohydrate fermentation to enhanced proteolytic fermentation in the ML group.

## 5. Conclusions

Our results indicated that fiber fraction, hydration properties, and fermentation are the primary characteristics distinguishing fiber raw materials. In vivo results showed that four fiber raw materials (PV, PG, MO, and ML), which differed in physicochemical properties, had distinctly divergent impacts on intestinal health and growth performance in meat rabbits. The key observations were that MO was associated with optimized cecal microbiota, increased SCFAs production, and lower diarrhea incidence; by contrast, despite its high in vitro fermentation, ML was associated with intestinal inflammation, reduced SCFAs, ADG, and ADFI, as well as elevated BCFAs, NH_3_-N, and diarrhea rate in vivo. Correlation analysis further revealed that enriched *Bacteroides* was closely associated with reduced growth performance and impaired intestinal health. Overall, these findings highlight that in vitro fermentation parameters should not be directly extrapolated to predict in vivo physiological outcomes, as the two are not always equivalent. Notably, MO with moderate hydration properties and high fermentation may be more beneficial for optimizing growth performance and intestinal health in meat rabbits than ML with high fermentation alone.

## Figures and Tables

**Table 1:** Basic properties of fiber raw materials.

Items (%) ^1^	PV	PG	MO	ML	Garlic Skin	Konjac Flour	Sodium Alginate	Paper Mulberry Leaf Meal	Kelp Fiber	Alfalfa Meal	Soybean Straw	Bamboo Fiber
DM	98.34	90.52	93.34	90.81	85.55	93.11	88.85	89.75	94.98	91.59	90.71	92.12
CP	6.70	0.91	10.37	5.36	0.54	1.53	0.47	0.65	3.09	2.43	0.71	0.13
CF	26.50	30.54	27.27	20.71	31.72	45.78	-	15.65	31.52	22.54	34.20	57.21
NDF	55.23	55.25	25.75	31.89	45.32	55.66	-	17.23	41.52	45.40	50.25	81.06
ADF	38.41	22.42	17.13	15.58	15.41	12.47	-	15.10	21.12	34.71	47.12	58.12

^1^ PV, peanut vine meal; PG, *Pennisetum giganteum* meal; MO, *Moringa oleifera* leaf meal; ML, mulberry leaf meal; DM, dry matter; CP, crude protein; CF, crude fiber; NDF, neutral detergent fiber; ADF, acid detergent fiber. Data are mean values of 4 independent batches of samples.

**Table 2:** Hydration properties and fermentation characteristics of fiber raw materials from different sources.

Items ^1^	PV	PG	MO	ML	Garlic Skin	Konjac Flour	Sodium Alginate	Paper Mulberry Leaf Meal	Kelp Fiber	Alfalfa Meal	Soybean Straw	Bamboo Fiber
Hydration properties												
WBC (g/g)	3.11	3.61	4.85	6.91	1.21	12.35	1.65	7.99	2.81	5.55	1.21	0.91
WSC (mL/g)	1.55	2.45	2.55	3.15	1.69	10.04	8.42	3.89	6.31	3.00	2.13	1.21
Viscosity (mPa·s)	2.01	2.14	2.14	2.10	1.05	18.45	25.12	2.50	8.42	2.31	1.02	2.09
Fermentation characteristics												
Gas production												
GP48 (mL/g)	595.00	426.14	1393.97	823.24	369.38	1174.65	1265.33	645.69	1731.81	352.48	57.59	532.64
IR_0_ × 100	0.62	0.66	1.38	0.84	0.62	0.44	0.33	0.73	1.35	0.33	0.36	0.51
K (h − 1)	0.04	0.04	0.04	0.04	0.03	0.30	0.10	0.04	0.10	0.04	0.03	0.03
T½ (h)	31.00	5.50	24.00	24.00	3.00	12.00	18.00	24.00	24.00	5.50	2.00	9.50
Protein fermentation products												
NH_3_-N (mg/L)	19.99	23.45	19.50	21.90	20.45	18.68	16.04	21.79	19.46	20.61	20.55	13.87
VFA production												
Acetate (μg/mL)	14.94	51.54	65.09	93.02	71.02	107.86	151.09	53.96	141.19	56.97	88.29	26.13
Propionate (μg/mL)	16.23	23.11	42.03	32.16	24.68	60.36	37.92	18.70	73.07	21.61	27.88	7.36
Butyrate (μg/mL)	16.07	10.92	8.46	10.02	10.20	15.19	12.49	6.25	9.05	12.97	15.81	3.63
Valerate (μg/mL)	15.92	2.13	1.67	1.43	1.85	2.26	1.12	0.64	1.63	1.99	1.41	0.83
Total SCFAs (μg/mL)	63.16	87.71	117.24	136.63	107.75	185.68	202.62	79.55	224.94	93.55	133.40	37.96
Isobutyrate (μg/mL)	16.71	3.29	2.33	2.83	2.89	1.79	1.51	2.69	2.34	2.29	4.19	1.79
Isovalerate (μg/mL)	16.08	4.27	2.29	3.09	3.57	1.53	1.39	2.82	0.68	2.62	4.64	1.45
Total BCFAs (μg/mL)	32.79	7.56	4.62	5.92	6.456	3.32	2.90	5.51	3.02	4.92	8.84	2.63
Total VFAs (μg/mL)	90.95	95.27	121.87	142.55	114.21	188.99	205.52	85.07	227.96	98.47	142.24	40.59
SCFAs/VFAs (%)	65.82	92.06	96.20	95.85	94.35	98.24	98.59	93.52	98.67	95.01	93.79	93.51

^1^ PV, peanut vine meal; PG, *Pennisetum giganteum* meal; MO, *Moringa leaf* meal; ML, mulberry leaf meal; WBC, water binding capacity; WSC, water swelling capacity; GP48, 48 h cumulative gas production; IR_0_, initial fermentation rate; K, fractional rate of gas production at a particular time point; T½, half-life to asymptote; NH_3_-N, ammonia nitrogen; VFAs, volatile fatty acids; SCFAs, short-chain fatty acids; BCFAs, branched-chain fatty acids. SCFAs are defined as the sum of acetate, propionate, butyrate, and valerate. BCFAs are defined as the sum of isobutyrate and isovalerate. Total VFAs are the sum of SCFAs and BCFAs. Data are mean values of 4 independent batches of samples.

**Table 3:** Spearman correlation coefficients between in vitro hydration properties and fermentation characteristics of 12 fiber raw materials ^1^.

Spearman Correlation	WBC (g/g)	WSC (mL/g)	Viscosity (mPa·s)
Gas production			
GP48 (mL)	0.26	0.47	0.58 *
IR_0_ × 100	0.09	−0.17	−0.13
K	0.56	0.83 **	0.57
T½ (h)	0.20	0.13	0.29
NH_3_-N (mg/L)	0.21	−0.07	−0.55
Volatile fatty acids			
Acetate (μg/mL)	0.25	0.68 *	0.34
Propionate (μg/mL)	0.46	0.61 *	0.18
Butyrate (μg/mL)	0.37	0.12	−0.38
Valerate (μg/mL)	0.66 *	0.02	−0.39
Total SCFAs (μg/mL)	0.38	0.67 *	0.25
Isobutyrate (μg/mL)	−0.09	−0.58	−0.84 **
Isovalerate (μg/mL)	0.01	−0.54	−0.84 **
Total BCFAs (μg/mL)	0.07	−0.43	−0.87 **
Total VFAs (μg/mL)	0.41	0.61 *	0.21
SCFAs/VFAs (%)	0.36	0.75 **	0.46
**Partial Spearman Correlation**	**WBC (g/g)**	**WSC (mL/g)**	**Viscosity (mPa·s)**
Gas production			
GP48 (mL)	−0.55	−0.14	−0.34
IR_0_ × 100	−0.84 *	−0.60	−0.77 *
K	0.75 *	0.89 *	0.97 **
T½ (h)	−0.16	−0.64	−0.61
NH_3_-N (mg/L)	−0.07	0.09	0.03
Volatile fatty acids			
Acetate (μg/mL)	−0.28	0.52	0.34
Propionate (μg/mL)	−0.38	0.48	0.32
Butyrate (μg/mL)	0.23	0.68 *	0.71 *
Valerate (μg/mL)	−0.13	−0.38	−0.16
Total SCFAs (μg/mL)	−0.30	0.66 *	0.39
Isobutyrate (μg/mL)	−0.20	−0.37	−0.25
Isovalerate (μg/mL)	−0.11	0.42	−0.20
Total BCFAs (μg/mL)	−0.15	−0.42	−0.21
Total VFAs (μg/mL)	−0.33	0.50	0.37
SCFAs/VFAs (%)	−0.12	0.56	0.35

^1^ WBC, water binding capacity; WSC, water solubility capacity; GP48, cumulative gas production at 48 h; IR0, initial gas production rate; K, gas production rate constant; T1/2, half-time of gas production; NH_3_-N, ammonia nitrogen; SCFAs, short-chain fatty acids; BCFAs, branched-chain fatty acids; VFAs, volatile fatty acids. Partial Spearman correlation coefficients between in vitro hydration properties and fermentation characteristics of 12 fiber raw materials, after controlling for dry matter, crude protein, and fiber fractions. The results in Table 3 are presented as correlation coefficients, where * indicates (*p* < 0.05) and ** indicates (*p* < 0.01).

**Table 4:** Eigenvalues and variance contribution rates of principal component analysis (PCA).

Items	Principal Component		
	1 (NDF, ADF, CF, Viscosity)	2 (WBC, WSC, GP48h and VFA)	3 (CP, DM, and NH_3_-N)
Initial eigenvalue	3.5	3.3	1.7
Percentage of variance (%)	35.8	29.8	15.1
Accumulation (%)	35.8	65.6	80.7

**Table 5:** Four representative dietary fibers with similar viscosity but distinct hydration properties and fermentation characteristics.

Items ^1^	PV	PG	MO	ML	SEM	*p* Value
WBC (g/g)	3.11 ^d^	3.61 ^c^	4.85 ^b^	6.91 ^a^	0.28	<0.001
WSC (mL/g)	1.55 ^c^	2.45 ^b^	2.55 ^b^	3.15 ^a^	0.16	<0.001
Viscosity (mPa·s)	2.01	2.14	2.14	2.30	0.04	0.083
Total VFAs (μg/mL)	90.95 ^c^	95.27 ^c^	121.87 ^b^	142.55 ^a^	5.62	<0.001

^1^ PV, peanut vine; PG, *Pennisetum giganteum* meal; MO, *Moringa oleifera* leaf meal; ML, mulberry leaf meal; WBC, water binding capacity; WSC, water swelling capacity; SCFAs, short-chain fatty acids. Means within the same row with different superscript letters are significantly different at *p* < 0.05.

**Table 6:** Composition of experimental diets.

Items ^1^	PV	PG	MO	ML
Ingredient (%)				
Corn	32.00	34.00	32.20	31.50
Soybean meal	22.00	22.00	21.80	21.50
Wheat bran	13.00	11.00	13.00	13.50
Fish meal	3.00	3.00	1.50	3.50
Wheat middlings	-	-	1.50	-
PV	25.00	-	-	-
PG	-	25.00	-	-
MO	-	-	25.00	-
ML	-	-	-	25.00
CaHPO_4_	0.50	0.50	0.50	0.50
Premix ^2^	4.50	4.50	4.50	4.50
Total	100	100	100	100
Calculated values				
Crude protein (%)	15.89	15.85	15.85	15.83
Crude fiber (%)	9.70	10.55	9.91	9.27
Neutral detergent fiber (%)	26.68	24.98	19.32	20.39
Acid detergent fiber (%)	13.81	9.27	8.47	7.91
Acid detergent lignin (%)	3.15	3.41	1.81	2.66
Soluble dietary fiber (%)	6.01	5.74	8.06	8.58
Digestible energy (MJ/kg)	10.66	10.96	10.98	10.63
Analyzed values				
Crude protein (%)	15.89	15.86	15.91	15.90
Crude fiber (%)	10.78	11.20	10.89	11.19
Neutral detergent fiber (%)	20.44	21.62	19.62	18.78
Acid detergent fiber (%)	14.05	14.32	13.46	13.37
Acid detergent lignin (%)	3.07	3.76	1.84	2.72
Soluble dietary fiber (%)	6.03	5.82	8.11	8.64

^1^ PV, peanut vine meal; PG, *Pennisetum giganteum* meal; MO, *Moringa oleifera* leaf meal; ML, mulberry leaf meal. All four fiber raw materials were included at 25% on a dry matter basis. The actual as-fed inclusion levels of the fiber sources and all other ingredients were calculated individually according to their respective dry matter contents, and the diets were then adjusted to be isonitrogenous and isocaloric. Notably, the as-fed inclusion levels were calculated individually based on the dry matter content of each fiber source: PV 25.42%, PG 27.62%, MO 26.78%, and ML 27.53%. ^2^ The composition of the premix includes vitamin A acetate, vitamin D_3_, vitamin E, vitamin K_3_, vitamin B_1_, vitamin B_2_, calcium D-pantothenate, nicotinamide, vitamin B_5_, D-biotin, folic acid, vitamin B_12_, choline chloride, ferrous sulfate monohydrate, copper sulfate pentahydrate, manganese sulfate monohydrate, zinc sulfate monohydrate, 1% sodium selenite premix, 1% potassium iodide premix, tricalcium phosphate, L-lysine monohydrochloride, DL-methionine, and sodium chloride. The carrier is rice bran.

**Table 7:** Effects of dietary fiber with different physicochemical properties on growth performance of New Zealand white rabbits.

Groups ^1^	PV	PG	MO	ML	SEM	*p* Value
No. of replicates	6	6	6	6		
Initial body weight (kg)	1.33	1.24	1.24	1.26	0.05	0.524
Final body weight (kg)	2.42 ^a^	2.14 ^b^	2.31 ^ab^	2.09 ^b^	0.07	0.012
ADFI (g)	108.03 ^a^	107.05 ^a^	106.77 ^a^	89.02 ^b^	9.66	<0.001
ADG (g) ^2^	31.25 ^a^	25.89 ^b^	30.53 ^a^	23.74 ^b^	2.07	0.045
F/G	3.52	4.41	3.52	4.24	0.33	0.243
Diarrhea rate (%) ^3^	16.67 ^ab^	12.50 ^ab^	8.30 ^b^	37.50 ^a^	-	0.041

^1^ PV, peanut vine meal; PG, *Pennisetum giganteum* meal; MO, *Moringa oleifera* leaf meal; ML, mulberry leaf meal; ADFI, average daily feed intake; ADG, average daily gain; F/G, feed-to-gain ratio. Values are presented as means ± standard error of the mean (SEM). Means within the same row with different superscript letters are significantly different at *p* < 0.05. ^2^ ADG was analyzed using the Kruskal–Wallis’s test. ^3^ Diarrhea incidence data were analyzed using a binomial GLM with logit link; the cage was the experimental unit. Overall treatment effect: likelihood-ratio χ^2^ = 8.27, *df* = 3. Pairwise comparisons: Fisher’s protected LSD test.

**Table 8:** Effects of dietary fiber with different physicochemical properties on the organ development of New Zealand white rabbits.

Groups ^1^	PV	PG	MO	ML	SEM	*p* Value
No. of rabbits	6	6	6	6		
Lung (g)	14.17	11.46	10.99	12.57	0.79	0.072
Heart (g)	6.95	6.19	6.47	5.72	0.37	0.163
Liver (g)	71.12	61.70	62.60	63.90	2.60	0.116
Spleen (g)	1.75	1.77	1.71	1.56	0.29	0.700
Kidney (g)	14.62	13.35	15.59	14.31	0.58	0.086
Round sac (g)	2.70	2.67	2.81	2.46	0.27	0.843
Appendix (g) ^2^	11.31 ^a^	9.58 ^ab^	10.75 ^a^	8.44 ^b^	0.75	0.040
Lung index (%)	0.58	0.54	0.48	0.59	0.13	0.062
Heart index (%)	0.29	0.29	0.28	0.28	0.01	0.929
Liver index (%)	2.95	2.89	2.76	3.04	0.11	0.315
Spleen index (%)	0.07	0.08	0.07	0.08	0.01	0.601
Kidney index (%)	0.61	0.62	0.68	0.68	0.02	0.055
Round sac index (%)	0.11	0.12	0.12	0.12	0.01	0.878
Appendix index (%)	0.47	0.45	0.47	0.40	0.03	0.383
Half-eviscerated carcass weight (kg)	1.63 ^a^	1.40 ^bc^	1.56 ^ab^	1.35 ^c^	0.07	0.016
Eviscerated carcass weight (kg)	1.52 ^a^	1.29 ^b^	1.45 ^ab^	1.27 ^b^	0.07	0.028
Dressing percentage (%)	62.66	60.20	62.77	60.38	1.57	0.565

^1^ PV, peanut vine meal; PG, *Pennisetum giganteum* meal; MO, *Moringa oleifera* leaf meal; ML, mulberry leaf meal. Means within the same row with different superscript letters are significantly different at *p* < 0.05. ^2^ Appendix was analyzed using the Kruskal–Wallis’s test; the remaining parameters were subjected to one-way analysis of variance (ANOVA) followed by Duncan’s multiple range test.

**Table 9:** Effects of dietary fiber with different physicochemical properties on meat quality of New Zealand white rabbits.

Groups ^1^	PV	PG	MO	ML	SEM	*p* Value
No. of rabbits	6	6	6	6		
Longissimus dorsi muscle						
Drip loss (g)	0.32	0.36	0.35	0.27	0.06	0.296
Shear force (N)	43.20	56.07	51.92	42.36	4.70	0.152
Single muscle fiber cross-sectional area (μm^2^)	1888.6	1927.62	1974.03	1686.76	123.46	0.428
Quadriceps femoris muscle						
Drip loss (g)	0.41	0.27	0.24	0.21	0.07	0.296
Shear force (N)	52.38 ^a^	42.69 ^ab^	35.15 ^b^	52.47 ^a^	4.73	0.043
Single muscle fiber cross-sectional area (μm^2^)	1790.27	2080.47	2221.65	2146.66	175.84	0.382

^1^ Means within the same row with different superscript letters (a, b, ab) are significantly different at *p* < 0.05, based on one-way ANOVA followed by Duncan’s multiple range test. The overall *p* values represent the treatment effect. PV, peanut vine; PG, *Pennisetum giganteum* meal; MO, *Moringa oleifera* leaf meal; ML, mulberry leaf meal.

**Table 10:** Effects of dietary fiber with different physicochemical properties on the serum physiology and biochemistry of New Zealand white rabbits.

Groups ^1^	PV	PG	MO	ML	SEM	*p* Value
No. of rabbits	6	6	6	6		
Glycolipid metabolism						
Glucose (mmol/L)	3.66	3.95	3.89	3.32	2.55	0.452
Triglyceride (mmol/L)	0.71	0.83	0.68	0.71	0.11	0.756
High-density lipoprotein (mmol/L)	0.60	0.31	0.59	0.28	0.12	0.227
Low-density lipoprotein (mmol/L)	0.39	0.30	0.29	0.35	0.07	0.741
Total cholesterol (mmol/L)	3.09	2.99	3.14	2.80	0.12	0.218
Free fatty acid (mmol/L)	0.43	0.47	0.49	0.43	0.04	0.562
Nitrogen metabolism						
Blood urea nitrogen (mmol/L)	4.61	4.42	4.74	4.71	0.29	0.870
Antioxidant status						
Malondialdehyde (mmol/mL)	2.82	2.12	2.14	2.08	0.36	0.641
Total antioxidant capacity (U/mL)	3.16	1.40	1.97	1.76	0.53	0.274
Intestinal inflammation						
Tumor necrosis factor-α (pg/mL)	2.62 ^b^	2.81 ^b^	2.77 ^b^	4.12 ^a^	0.41	<0.001
D-Lactate (mmol/L)	0.17 ^bc^	0.19 ^b^	0.12 ^c^	0.24 ^a^	0.09	0.048
Diamine oxidase (U/L)	1.24 ^c^	1.52 ^bc^	1.23 ^c^	2.01 ^a^	0.25	0.026
Lipopolysaccharide (EU/mL)	0.18 ^b^	0.24 ^a^	0.23 ^a^	0.21 ^ab^	0.01	0.014
Lipopolysaccharide-binding protein (μg/mL) ^2^	4.21	5.18	5.33	5.36	0.20	0.151

^1^ Means within the same row with different superscript letters (a, b, c, ab, bc) are significantly different at *p* < 0.05, based on one-way ANOVA followed by Duncan’s multiple range test. The overall *p* values represent the treatment effect. PV, peanut vine; PG, *Pennisetum giganteum* meal; MO, *Moringa oleifera* leaf meal; ML, mulberry leaf meal. ^2^ For lipopolysaccharide-binding protein only, one outlier in PV group (8.50 μg/mL) was excluded (>mean ± 3 SD); n = 5 for PV-LBP, n = 6 for all others.

**Table 11:** Effects of dietary fiber with different physicochemical properties on the intestinal morphology of the duodenum and cecum of New Zealand white rabbits.

Groups ^1^	PV	PG	MO	ML	SEM	*p* Value
No. of rabbits	6	6	6	6		
Duodenum						
Villus height (μm)	786.24	976.57	789.43	823.45	160.44	0.209
Crypt depth (μm)	235.99	294.81	265.32	257.76	66.81	0.588
Villus height/crypt depth	3.40	3.33	3.01	3.43	0.59	0.733
Cecum						
Mucosal depth (μm)	524.42 ^b^	385.27 ^c^	493.75 ^b^	633.64 ^a^	29.46	<0.001
Intestinal wall thickness (μm)	1683.23 ^ab^	1470.77 ^b^	1496.21 ^b^	1886.72 ^a^	81.30	0.011
Cecal histological score	1.33 ^a^	1.00 ^a^	1.50 ^a^	2.83 ^b^	0.23	<0.001

^1^ Means within the same row with different superscript letters (a, b, c, ab) are significantly different at *p* < 0.05, based on one-way ANOVA followed by Duncan’s multiple range test. The overall *p* values represent the treatment effect. PV, peanut vine meal; PG, *Pennisetum giganteum* meal; MO, *Moringa oleifera* leaf meal; ML, mulberry leaf meal.

**Table 12:** Concentrations of volatile fatty acids and ammonia nitrogen in the cecal contents of growing rabbits fed diets supplemented with different fiber raw materials.

Groups ^1^	PV	PG	MO	ML	SEM	*p* Value
No. of rabbits	6	6	6	6		
Acetate (μg/mg)	4.13 ^b^	4.54 ^b^	6.18 ^a^	2.64 ^c^	0.32	0.003
Propionate (μg/mg)	1.16 ^b^	1.24 ^b^	2.45 ^a^	0.71 ^c^	0.14	0.002
Butyrate (μg/mg)	0.57 ^bc^	0.76 ^b^	1.31 ^a^	0.51 ^c^	0.09	0.011
Valerate (μg/mg)	0.14 ^bc^	0.16 ^ab^	0.21 ^a^	0.09 ^c^	0.02	0.037
Total SCFAs (μg/mg)	6.30 ^b^	6.90 ^b^	9.46 ^a^	3.97 ^c^	0.45	<0.001
Isobutyrate (μg/mg)	0.01 ^c^	0.09 ^bc^	0.08 ^b^	0.16 ^a^	0.02	0.029
Isovalerate (μg/mg)	0.13 ^b^	0.11 ^bc^	0.10 ^c^	0.22 ^a^	0.02	0.042
Total BCFAs (μg/mg)	0.23 ^b^	0.20 ^bc^	0.18 ^c^	0.38 ^a^	0.03	0.031
Total VFAs (μg/mg)	6.53 ^b^	7.10 ^b^	9.64 ^a^	4.35 ^c^	0.47	<0.001
SCFAs/VFAs (%)	90.48 ^b^	91.18 ^b^	98.13 ^a^	91.26 ^b^	1.12	0.025
NH_3_-N (μg/g)	257.15 ^b^	236.54 ^bc^	245.78 ^c^	299.68 ^c^	10.45	0.001

^1^ PV, peanut vine meal; PG, *Pennisetum giganteum* meal; MO, *Moringa oleifera* leaf meal; ML, mulberry leaf meal; SCFAs, short-chain fatty acids; BCFAs, branched-chain fatty acids; VFAs, volatile fatty acids. ^a–c^ Means with different superscripts within a row differ significantly (*p* < 0.05).

## Data Availability

All data generated or analyzed during this study are included in the article/Supplementary Materials. Further information is available from the corresponding author.

## Supplementary Materials

The following supporting information can be downloaded at https://www.mdpi.com/article/10.3390/ani16182921/s1. Table S1. Dietary fiber raw materials, commercial suppliers, and item numbers.

## Abbreviations

The following abbreviations are used in this manuscript:

ADF	Acid detergent fiber
ADFI	Average daily feed intake
ADG	Average daily gain
ANOVA	Analysis of variance
BCFAs	Branched-chain fatty acids
CF	Crude fiber
CP	Crude protein
DM	Dry matter
F/G	Feed-to-gain ratio
GP48	Cumulative gas production at 48h
IR0	Initial gas production rate
K	Gas production rate constant
LEfSe	Linear discriminant analysis effect size
MO	*Moringa oleifera* leaf meal
ML	Mulberry leaf meal
NDF	Neutral detergent fiber
NH_3_-N	Ammonia nitrogen
PCA	Principal component analysis
PCoA	Principal coordinate analysis
PG	*Pennisetum giganteum* meal
PV	Peanut vine meal
SCFAs	Short-chain fatty acids
SEM	Standard error of the mean
T½	Half-time of gas production
VFAs	Volatile fatty acids
WBC	Water binding capacity
WSC	Water solubility capacity
