# Supplementary material for: Effects of Dietary Fibers with Different Physicochemical Properties on Intestinal Health and Growth Performance of Meat Rabbits
Source: Animals (Basel). 2026 Sep 17;16(18):2921. doi: 10.3390/ani16182921 (PMC13603262; doi:10.3390/ani16182921)
Supplement: Supplementary file 1 [file animals-16-02921-s001.zip › animals-4522748-supplementary.pdf]

Supplementary Table S1. Dietary fiber raw materials, commercial suppliers, and item numbers.

Dietary fiber raw materials	Suppliers	Item number
PV	Zhengyang Xintiandi Grass Industry Co., Ltd.	unavailable
PG	National Engineering Research Center of Juncao Technology	unavailable
MO	Shaanxi Haochen Biotechnology Co., Ltd.	h4578
ML	Xinyang Mufan Biotechnology Co., Ltd.	MF-000927
Garlic skin	Shandong Jinan Limin Corncob Products Business Department	unavailable
Konjac flour	Jiangsu Congzhong Chemical Co., Ltd.	30232
Sodium alginate	Zhengzhou Chengwang Chemical Products Co., Ltd.	158
Paper mulberry leaf meal	Shaanxi Sinuote Biotechnology Co., Ltd.	unavailable
Kelp fiber	Xinyang Mufan Biotechnology Co., Ltd.	MF-013309
Alfalfa meal	Baoji Fangsheng Biological Development Co., Ltd.	LS-6666
Soybean straw	Shandong Hongda Grass Industry Bean Straw Powder Development Co., Ltd.	unavailable
Bamboo fiber	Xinyang Mufan Biotechnology Co., Ltd.	MF-013325
